# Potential Benefits of In Silico Methods: A Promising Alternative in Natural Compound’s Drug Discovery and Repurposing for HBV Therapy

**DOI:** 10.3390/ph18030419

**Published:** 2025-03-16

**Authors:** Samuel Chima Ugbaja, Aganze Gloire-Aimé Mushebenge, Hezekiel Kumalo, Mlungisi Ngcobo, Nceba Gqaleni

**Affiliations:** 1 Discipline of Traditional Medicine, School of Nursing and Public Health, University of KwaZulu Natal, Durban 4000, South Africa; ngcobom3@ukzn.ac.za; 2 Department of Pharmacology, University of the Free State, Bloemfontein Campus, Bloemfontein 9301, South Africa; aganzedar@gmail.com; 3 Faculty of Pharmaceutical Sciences, University of Lubumbashi, Lubumbashi 1825, Democratic Republic of the Congo; 4 Drug Research and Innovation Unit, Discipline of Medical Biochemistry, School of Laboratory Medicine and Medical Science, University of KwaZulu-Natal, Durban 4000, South Africa; kumaloh@ukzn.ac.za

**Keywords:** natural compounds, in silico methods, drug discovery, drug repurposing, HBV therapy

## Abstract

Hepatitis B virus (HBV) is an important global public health issue. The World Health Organization (WHO) 2024 Global Hepatitis Report estimated that the global prevalence of people living with HBV infection is 254 million, with an estimated prevalence incidence of 1.2 million new HBV infections yearly. Previous studies have shown that natural compounds have antiviral inhibition potentials. In silico methods such as molecular docking, virtual screening, pharmacophore modeling, quantitative structure–activity relationship (QSAR), and molecular dynamic simulations have been successfully applied in identifying bioactive compounds with strong binding energies in HBV treatment targets. The COVID-19 pandemic necessitated the importance of repurposing already approved drugs using in silico methods. This study is aimed at unveiling the benefits of in silico techniques as a potential alternative in natural compounds’ drug discovery and repurposing for HBV therapy. Relevant articles from PubMed, Google Scholar, and Web of Science were retrieved and analyzed. Furthermore, this study comprehensively reviewed the literature containing identified bioactive compounds with strong inhibition of essential HBV proteins. Notably, hesperidin, quercetin, kaempferol, myricetin, and flavonoids have shown strong binding energies for hepatitis B surface antigen (HBsAg). The investigation reveals that in silico drug discovery methods offer an understanding of the mechanisms of action, reveal previously overlooked viral targets (including PreS1 Domain of HBsAg and cccDNA (Covalently Closed Circular DNA) regulators, and facilitate the creation of specific inhibitors. The integration of in silico, in vitro, and in vivo techniques is essential for the discovery of new drugs for HBV therapy. The insights further highlight the importance of natural compounds and in silico methods as targets in drug discovery for HBV therapy. Moreover, the combination of natural compounds, an in silico approach, and drug repurposing improves the chances of personalized and precision medicine in HBV treatment. Therefore, we recommend drug repurposing strategies that combine in vitro, in vivo, and in silico approaches to facilitate the discovery of effective HBV drugs.

## 1. Introduction

The hepatitis B virus (HBV) remains a significant worldwide health issue, leading to both acute and chronic liver conditions like fibrosis, cirrhosis, and hepatocellular carcinoma (HCC) [1,2]. Although there is a preventive vaccine, HBV continues to infect millions each year, and chronic infections lead to considerable illness and death [2,3,4]. According to the World Health Organization (WHO) 2024 Global Hepatitis Report, the global prevalence of people living with HBV infection is estimated at 254 million, with an approximated incidence prevalence of 1.2 million new HBV infections per annum [5]. The persistence of HBV in hepatocytes, largely due to covalently closed circular DNA (cccDNA), plays a role in its resistance against existing antivirals that focus on reverse transcription yet do not eradicate this viral reservoir [6,7]. Existing treatments, such as interferon-alpha and nucleos(t)ide analogs, face challenges due to drug resistance and failure to eliminate the infection, underscoring the necessity for new therapeutic approaches [8,9]. Previous studies have shown that the stable viral reservoir potential of cccDNA enhances HBV resistance and persistence regardless of antiviral treatment. HBV resistance is achieved via epigenetic modifications, ineffective direct degradation of cccDNA by the nucleotide and nucleoside analogs, and the low immune system of the patient. Moreover, the efficacy of HBV therapy is limited by the mutation of HBV polymerase, resulting in drug resistance. This further strengthens the need for exploring an in silico-focused drug repositioning and natural product alternative [10,11].

Natural compounds, such as phenylpropanoids, flavonoids, and terpenoids, have previously shown potential effects of antiviral inhibition by disrupting the viral lifecycle, including replication and interactions specific to the host [12]. By employing various computational techniques, researchers have successfully examined extensive libraries of bioactive compounds and identified molecular traits crucial for antiviral efficacy, accelerating the identification of new therapeutic agents [13]. 

**Figure 1:**
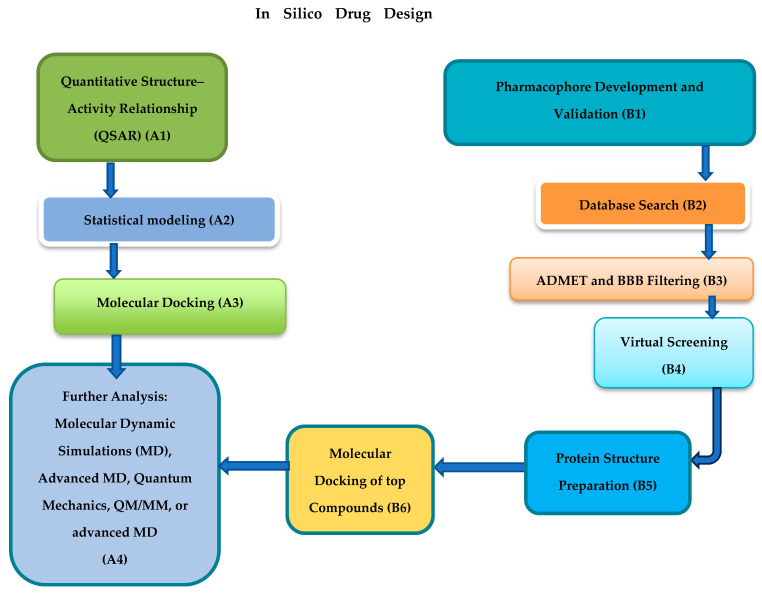
Diagram illustration of in silico Drug Design Methods.

Progress in computational drug discovery, such as homology modeling, molecular docking, and dynamics simulations (Figure 1), provides promising options for finding new drug candidates and repurposing current ones for HBV therapy [14]. These tools offer essential insights into interactions of viral proteins, targets for drugs, and the nature of drug binding, facilitating the discovery of compounds that demonstrate strong efficacy and safety profiles [14,15]. When used alongside conventional pharmacological research, these in silico techniques improve the capacity to create targeted treatments that could ultimately result in the elimination of HBV [16,17]. The aim of this review is to unveil the benefits of in silico techniques as an alternative to natural compounds’ drug discovery and repurposing for HBV therapy.

In silico drug design could be processed via two different routes (A and B), as depicted in Figure 1 above. Pharmacophore screening, development, and validation (B1) and database exploration (B2) are carried out to discover compounds or drug candidates that share structural similarities with the natural ligand or substrate of the protein (receptor). Afterward, compounds undergo absorption, distribution, metabolism, excretion, and toxicity (ADMET) and blood–brain barrier (BBB) filtering analyses to select drug candidates with advantageous pharmacokinetic characteristics (B3) [18,19]. Virtual screening is subsequently utilized to quickly assess extensive compound libraries for their potential to bind to the receptor (B4). Protein structure preparation (B5) is followed by molecular docking (B6) to forecast the binding mode and affinity of small molecules to the receptor [20,21,22,23,24]. Conversely, QSAR modeling is employed to create mathematical connections between chemical structures and biological activity (A1–A2), helping to identify possible drug candidates [25,26]. Molecular docking forecasts how ligands bind and their affinities target proteins by analyzing ligand shapes and assessing optimal binding arrangements (A3) [27]. The chosen candidates are subjected to molecular dynamics (MD) simulations to assess binding affinity and stability, after which the binding energy is predicted using the Generalized Born Surface Area (MM-GBSA) solvation method. This thorough method provides an effective way to screen extensive compound libraries or repurposed medications, thereby yielding valuable information on possible drug efficacy prior to experimental evaluation [28].

The obstacles of traditional drug discovery, such as elevated expenses, extended durations, and significant failure rates, have driven a movement toward different approaches, including drug repurposing [29,30]. Computational tools are speeding up the discovery of existing medications that focus on HBV-specific pathways, like cccDNA regulation [31]. Drug repurposing utilizes established safety profiles and action mechanisms, decreasing development duration and expenses while tackling HBV’s resistance to conventional treatments [32]. Furthermore, combining computational methods with conventional medicine presents new opportunities for HBV therapy. Herbal formulations such as Liujunzi decoction (LJZD) and Xiaochaihu decoction (XCHD) are currently under investigation for their multitarget therapeutic mechanisms through network pharmacology and molecular modeling [33,34,35]. These investigations pinpoint essential compounds, including quercetin and chrysin, that engage with HBV-related targets, further reinforcing the promise of traditional medicine (natural compounds) in contemporary antiviral drug creation [34,35]. Integrating computational methods with ethnopharmacological studies enhances our understanding of HBV pathogenesis and uncovers novel therapeutic approaches that can be tested in both in vitro and in vivo environments. The synergy between computational chemical biology, bioinformatics, and conventional medicine offers significant potential for creating effective and safe therapies for HBV. Utilizing sophisticated modeling strategies and AI, scientists can enhance drug discovery methods, increasing the effectiveness, safety, and scalability of antiviral therapies [36,37].

## 2. Reasons for Exploring in Silico Drug Design in Repurposing, Especially for HBV Treatment

HBV continues to be a significant worldwide health issue, even with the existence of a preventive vaccine [38,39]. Current therapies approved by the Food and Drug Administration (FDA), such as interferons and nucleos(t)ide analogs, mainly lower antigen levels but do not eliminate the virus [40]. Recent developments, including the discovery of capsid assembly modulators (CAMs), show encouraging antiviral potential [41]. Nevertheless, creating new HBV inhibitors demands creative approaches to address the shortcomings of existing methods [42]. In silico or computational methods such as molecular docking, pharmacophore modeling, and molecular dynamics simulations have demonstrated their effectiveness in designing novel inhibitor compounds. Computational drug discovery tools enable researchers to effectively screen vast numbers of compounds and prioritize those with beneficial antiviral characteristics, greatly accelerating the drug discovery process.

The effectiveness of computational techniques is clear from their capacity to tackle problems encountered in antiviral studies for viruses such as the hepatitis C virus (HCV). The development of HCV medications has gained from structure-based drug design and virtual screening to address challenges such as drug resistance and insufficient specificity [43,44]. Structure-based drug design (SBDD) has been useful in the development of new drugs. The three-dimensional (3-D) structures, x-ray crystallography, or NMR spectroscopy of the target proteins were retrieved from the Research Collaboratory for Structural Bioinformatics Protein Data Bank (RCSB-PDB). The process included exploring compound databases and a library of compounds such as ChemSpider, PubChem, and ChEMBL for data collection. Thereafter, virtual screening and optimization using the Schrodinger software suite were performed. Subsequent molecular docking and molecular dynamic simulations were performed using maestro Glide and Desmond, respectively. Notably, drug compounds developed using SBDD interacted with the target protein and reduced the incidence of side effects while improving the efficacy of the therapy. The discovery of new drugs requires the complementarity of SBDD and ligand-based drug design in determining and analyzing the 3-D structures of bioactive compounds. Other databases that specialize in commercially available compounds and natural compounds include the Dictionary of Natural Products (DNP), Super Natural II, Universal Natural Products Database (UNPD), and ZINC databases were also explored [45,46]. These approaches offer an understanding of the action mechanisms, reveal previously overlooked viral targets, and facilitate the creation of specific inhibitors [47]. In a similar way, in silico methods for HBV can help discover new CAMs and other therapeutic agents by forecasting drug–target interactions and refining lead compounds [48]. These methods provide an economical and time-saving framework to investigate and enhance drug candidates, meeting the pressing demand for effective HBV therapies.

Additionally, the COVID-19 pandemic has underscored the importance of repurposing current medications through computational methods. Molecular docking and pharmacogenetic evaluations have expedited the discovery of possible treatments by utilizing established pharmacokinetic and pharmacodynamic characteristics [49]. Implementing these insights into HBV treatment can improve precision medicine by recognizing synergistic combinations and reducing negative interactions. Moreover, combining computational techniques with conventional medicine and natural product evaluation creates new pathways for identifying bioactive substances. In silico methods not only facilitate drug development by closing gaps in experimental research but also enhance our comprehension of viral pathogenesis and therapeutic strategies. Table 1 below summarizes research efforts toward drug repurposing and natural compounds in HBV therapy achieved through in silico methods. 

## 3. Mechanistic Overview of In Silico Methods Employed for Drug Discovery

In silico methods seem to provide a revolutionary way to tackle issues in drug development for HBV, including resistance mutations and the inadequate effectiveness of existing treatments [83,84]. Molecular docking, essential to Computer-Aided Drug Design (CADD), estimates the binding orientation and affinity of ligands to the receptors (viral proteins). Docking aids in identifying lead compounds with strong antiviral potential by assessing interactions such as hydrogen bonding and hydrophobic forces [85]. For example, docking research on some substances targeting the HBV capsid protein GIPC2 identified a candidate compound with encouraging inhibitory effects [86]. Quantitative structure–activity relationship (QSAR) analysis enhances this process by linking molecular descriptors to bioactivity, allowing for the creation of analogs with improved therapeutic effects [86]. Molecular dynamics (MD) simulations enhance docking by investigating the stability and conformational adaptability of drug–protein interactions throughout time [87]. This dynamic method evaluates the interactions of compounds in physiological conditions, offering insights into the persistence of inhibitory effects [85,87]. In HBV studies, MD simulations have been employed to investigate resistance mutations and enhance interactions between possible inhibitors and viral proteins [88]. Consequently, homology modeling forecasts the configuration of viral proteins, bridging gaps in experimental information and directing virtual screening (VS) activities. These methods collectively allow for a structured assessment of both effectiveness and possible resistance mechanisms [15]. 

Furthermore, while computationally screening millions of compounds, researchers can prioritize those possessing beneficial drug-like characteristics for additional assessment [89]. For instance, a research project that merged virtual screening with IC_50_ forecasting recognized gallic acid derivatives as possible blockers of the HBV capsid protein [90,91]. ADMET (absorption, distribution, metabolism, excretion, and toxicology) evaluation guarantees that chosen compounds demonstrate advantageous pharmacokinetics and low toxicity, thereby optimizing the drug development process [92]. By combining these approaches, researchers can effectively pinpoint and enhance candidates with significant therapeutic potential. 

The investigation of innovative therapeutic techniques, such as the repurposing of antiretrovirals and the incorporation of traditional medicine, is also made easier by in silico approaches. By using their established pharmacological profiles, computational models can assess current medications against HBV targets and shorten development times [93,94]. Docking and QSAR investigations can be used to evaluate the antiviral activity of chemicals used in traditional medicine, such as quinines and natural products [95]. These methods advance the development of next-generation therapeutics by filling in gaps in therapeutic innovation and improving our understanding of HBV pathophysiology.

Previous in silico studies on a variety of plant-derived natural compounds with well-established antiviral properties have also shown a substantial binding affinity to hepatitis B surface antigen (HBsAg) and HBV polymerase. Some of the plant-derived natural compounds with relatively high binding antiviral affinity include glycyrrhizin, luteolin, and quercetin. Further studies have unraveled mechanisms and ways in which these substances interact with the human immune system, including their potential to enhance currently utilized antiviral treatments. Although these results offer a strong case for experimental validation, many of them are still in the preclinical stage, emphasizing the need for more in vitro and in vivo research [96,97,98,99]. More so, other studies have previously reported that only a small number of natural substances have made it to clinical trials despite multiple computational forecasts of promising HBV inhibitors. For example, compounds such as silibinin, silymarin, and berberine have been clinically evaluated for HBV therapy because of their hepatoprotective and antiviral potentials. These natural substances have demonstrated the ability to alter liver function and viral replication, highlighting the necessity of more translational studies to close the gap between clinical use and computer predictions [100,101,102,103]. Due to the limitations associated with some of the previously in silico studied natural compounds, including inefficient pharmacokinetics and poor bioavailability, additional optimization through drug formulation techniques, structural alterations, or nanoparticle-based delivery systems is required to maximize their therapeutic potential [104,105]. Other natural compounds that have demonstrated encouraging antiviral action against HBV include curcumin and andrographolide. However, because of their solubility, stability, and bioavailability limitations, their clinical translation is still difficult. Additional optimization through structural changes, sophisticated drug delivery methods, or synergistic combination therapy is required to increase their clinical viability [97,100,106,107,108,109].

## 4. Usefulness of Molecular Docking in Evaluating Binding Affinities and Interactions of Natural Compounds with HBV-Related Targets

Molecular docking analysis offers an essential understanding of how natural compounds and antiretrovirals (ARVs) interact with HBV targets, facilitating the discovery of promising therapeutic agents [94,110]. Viral infections such as hepatitis B remain major public health challenges because of drug resistance and restricted treatment effectiveness [109,110]. Natural products, abundant in a variety of structural compounds, have historically been used in traditional medicine for the treatment of viral infections [67,102]. Docking simulations of bioactive substances, such as hesperidin, quercetin, and kaempferol, have shown considerable interactions with HBV proteins, indicating their potential as antivirals [66,111]. For example, flavonoids present in Salicornia spp., such as myricetin and isoquercitrin, have demonstrated encouraging docking scores and inhibitory impacts on HBV targets, underscoring the promise of plant-based compounds in antiviral drug development [112]. 

The RNase H activity of HBV is an essential target for obstructing viral replication, and molecular docking analyses have recognized substances like α-hydroxytropolones and N-hydroxyisoquinolinediones as effective RNase H inhibitors [113,114]. These compounds interfere with the synchronized function of DNA polymerase and RNase H domains, essential for the synthesis of double-stranded viral DNA [115]. Likewise, substances derived from nature, such as EGCG (epigallocatechin-3-gallate) and curcumin, have demonstrated, via in silico techniques, the ability to inhibit various HBV molecular targets, offering both antiviral effects and minimizing toxicity [116,117]. Docking studies support the promise of these molecules as viable substitutes for synthetic antiviral medications. 

The investigation of medicinal plants like *Euphorbia schimperi* and *Cardiospermum halicacabum* has further propelled the discovery of HBV inhibitors [118]. Flavonols extracted from *E. schimperi*, quercetin-3-O-glucuronide (Q3G), quercetin-3-O-rhamnoside (Q3R), and kaempferol-3-O-glucuronide (K3G) demonstrated considerable inhibition of HBsAg and HBeAg production in cells infected with HBV [119,120]. Molecular docking analyses indicated that these compounds engage with HBV polymerase and core proteins, reinforcing their in vitro anti-HBV efficacy [120]. Likewise, methanol extracts from *C. halicacabum* showed inhibitory effects on both HIV reverse transcriptase and HBV surface antigen, highlighting the dual therapeutic possibilities of bioactive compounds obtained from plants [120,121]. 

Antiretroviral medications utilized for HIV therapy also demonstrate potential for repurposing against HBV because of the structural resemblances in their viral enzymes [122]. Inhibitors of RNase H, such as N-hydroxypyridinediones, demonstrate effectiveness against RNase H in both HIV and HBV, with docking simulations emphasizing their capacity to engage with common replication pathways [16,122,123]. This dual inhibitory function not only expands the treatment options of current medications but also shortens the development periods for HBV-targeted therapies [123,124]. These repurposing initiatives, bolstered by molecular docking and structural activity assessments, showcase a budget-friendly approach to tackling HBV treatment obstacles. 

Molecular docking research has highlighted the capabilities of natural compounds and repurposed ARVs in HBV therapy by clarifying their interactions with viral targets. These computational techniques facilitate the discovery of lead compounds that exhibit strong binding affinities and advantageous pharmacological characteristics. Through the combination of natural product research and drug repurposing initiatives, molecular docking is furthering the creation of novel and effective treatments for HBV, meeting the worldwide demand for better antiviral drugs. 

## 5. Pharmacophore Modeling Benefits in Identifying Natural Compound HBV Inhibitors

Pharmacophore modeling acts as an effective computational approach for discovering potential HBV inhibitors by forecasting the binding efficacy of natural and plant-sourced compounds with viral targets [125]. Hepatitis infections, especially those resulting from HBV, greatly affect liver health and are linked to chronic inflammation and liver cancer [126]. Natural substances, such as phenylpropanoids, flavonoids, and terpenoids, have demonstrated notable antiviral effects by disrupting different phases of the viral lifecycle, including replication and interactions specific to the host [10]. Employing pharmacophore models allows researchers to examine extensive libraries of bioactive compounds to identify molecular traits crucial for antiviral efficacy, accelerating the identification of new therapeutic agents. 

In silico research focused on the HBx protein, a key regulator in HBV pathogenesis and hepatocellular carcinoma, emphasizes the value of pharmacophore modeling [127]. Although a definitive crystal structure is absent, the application of computational methods has enabled the forecasting of HBx models, facilitating docking experiments with plant-derived substances [127]. For instance, rutin, a type of flavonoid, showed a significant binding energy of −161.65 kcal/mol with HBx, prompting the design and evaluation of rutin derivatives [127,128]. These initiatives recognized rutin01 and rutin08, showcasing enhanced binding energies of −163.16 and −165.76 kcal/mol, respectively, for additional experimental confirmation [127,128]. These methods highlight the ability of pharmacophore modeling to enhance candidate molecules by simulating their interactions with viral proteins, even in cases where structural data are scarce. 

The use of pharmacophore modeling goes beyond HBV to include other viral infections, including HCV, where compounds derived from plants have demonstrated inhibitory effects on essential entry proteins such as CD81 and CLDN1 [129,130]. Computational methods have been utilized to evaluate flavonoids like quercetin, taxifolin, and (-)-epicatechin, which show significant binding affinities to these receptors, as illustrated by molecular dynamics simulations and MM/GBSA free energy assessments [131]. These investigations not only underscore the importance of pharmacophore-oriented screening for discovering antiviral agents but also stress the necessity for further in vitro and in vivo validation to convert computational results into clinical use. Pharmacophore modeling, therefore, connects the fields of natural product discovery and antiviral drug development, providing an encouraging avenue for novel treatments for HBV and similar infections. 

## 6. QSAR and ADMET in Natural Compounds Bioactivity Predictions

Quantitative structure–activity relationship (QSAR) modeling serves as a crucial resource in drug development, providing an understanding of the connection between molecular structure and biological function. In the realm of HBV, QSAR analyses facilitate the organized assessment of natural substances for their possible antiviral properties [132,133]. Through the examination of structural characteristics and their relationship with experimental bioactivity, scientists can create more efficient molecules [41]. For example, QSAR modeling used on 79 HCV NS5B polymerase inhibitors identified essential molecular descriptors that affect binding affinity, leading to new candidates with enhanced bioactivity relative to reference drugs [134,135]. These models facilitate the pinpointing of structural components essential for focusing on viral proteins and promoting the progress of novel HBV treatments. 

Absorption, Distribution, Metabolism, Excretion, and Toxicity (ADMET) evaluation enhances QSAR by examining the pharmacokinetic and safety characteristics of prospective drug candidates. In HBV drug discovery, ADMET predictions assess the drug-like characteristics of natural compounds, confirming that the selected molecules possess favorable traits like bioavailability, minimal toxicity, and metabolic stability [129]. Recent research utilizing in silico ADMET tools has shown improved pharmacokinetics of newly developed compounds in comparison to existing antivirals. For instance, derivatives exhibiting enhanced binding energies against HCV NS3/A4 protease and NS5B polymerase demonstrated encouraging ADMET profiles, highlighting the usefulness of computational methods in preclinical drug assessment [136]. 

The combination of QSAR and ADMET predictions with molecular docking and machine learning speeds up the identification of potential therapeutic agents for viral infections [137]. In HCV studies, these methods revealed multiple high-affinity inhibitors, including compounds that focus on the ROCK1 enzyme, which is crucial for viral entry. Likewise, the integration of QSAR-ML and ADMET evaluation has identified compounds with drug-like properties and remarkable binding affinities, offering frameworks for HBV treatment [137,138,139]. By computationally refining lead compounds, these methods lessen the reliance on time-consuming laboratory experiments, establishing the foundation for the logical design of antiviral agents with enhanced efficacy and safety profiles. 

## 7. Future Perspectives and Research Directions

Computational chemical biology plays a critical role in HBV research through bioinformatics and first-principle methods, enabling insights into biomolecular interactions and drug resistance. Key techniques like homology modeling, molecular docking, and molecular dynamics predict protein structures, ligand interactions, and biomolecule stability. These methods have advanced understanding of HBV resistance mutations and viral genome analysis, aiding drug development and genotyping. As an essential tool in antiviral research, computational approaches are integral to developing new therapies for HBV. In a recent study by our group (Ugbaja S.C et al., 2025), we uniquely explored the intersection of pharmacogenetics and drug repurposing for optimized HBV therapy. This study recommended that in vivo, clinical trials, and in silico research are important for the validation of the potency, optimum dosage, and safety of repurposed antiretrovirals in HBV treatment. Further recommendations included the prioritization of research collaborations comprising regulators and funders to foster clinical adoption and incorporation of repurposed ARVs for HBV therapy. Therefore, integrating natural compounds and in silico drug discovery in addition to the previously recommended options will foster the development of drugs that would eliminate HBV [140,141].

## 8. Importance of Integrative In Silico, In Vitro, and In Vivo Methods in Drug Discovery

A comprehensive strategy that merges in silico, in vitro, and in vivo techniques is essential for enhancing the knowledge and management of HBV infections. Chronic HBV continues to be a worldwide health concern, leading to serious consequences like liver cirrhosis and hepatocellular carcinoma. The intricate nature of its pathogenesis and molecular mechanisms requires various research models. Although humanized animal models such as chimeric mice have greatly progressed HBV research by mimicking human-specific HBV interactions, they continue to be expensive and logistically complicated [140]. In vitro systems present an alternative by offering controlled settings to explore HBV replication processes and drug effectiveness, although they frequently lack the systemic intricacies of living organisms. Computational in silico models enhance these methods by allowing for quick simulation of molecular interactions and forecasting drug behaviors, such as risks of hepatotoxicity. This trio of approaches tackles the drawbacks found in any individual model, providing a more comprehensive and thorough comprehension of HBV biology and treatment reactions [140]. 

Furthermore, other in silico studies have revealed efficient HBV inhibition by employing pharmacophore modeling, molecular docking, and molecular dynamics simulations. For example, a study by Furutani Y. et al. (2023) identified a new small molecule named iCDM-34 (a pyrazole moiety) with the potential to effectively inhibit HBV using an in silico approach [142]. Another study by Elmessaoudi-Idrissi, Mohcine, et al. (2018) further elucidated our stance that the current HBV drugs exhibited limited potency for inhibiting HBV. The authors identified new potential HBV drugs using virtual screening and also stated that the combination of in silico and in vitro methods could enhance the validation of new drug targets for HBV therapy [143]. This further agrees with what we had recommended in our recently published study that “in vivo, clinical trials, and in silico research are important for validation of the potency, optimum dosage and safety of repurposed antiretrovirals in HBV treatment” [141].

Cutting-edge technologies like artificial intelligence (AI) and machine learning (ML) boost the predictive precision of integrative HBV research models [144]. These tools have demonstrated potential in enhancing in silico models for predicting hepatotoxicity and assessing drug-induced liver injury (DILI), which continue to be significant obstacles in drug development [92]. Furthermore, progress in microRNA (miRNA) studies provides fresh opportunities for personalized medicine since miRNAs are increasingly acknowledged for their regulatory functions in viral infections and their prospects as diagnostic markers and treatment targets [145]. New approaches, such as gene vaccines and focused antiviral therapies for at-risk groups like pregnant women, highlight the importance of precision and safety [47]. By combining computational forecasts, experimental confirmations, and practical applications, future HBV studies can hasten the creation of effective treatments while reducing negative effects. This comprehensive approach promotes advancements in tackling HBV and enhancing results in varied clinical settings. 

## 9. Potential Role of AI and Machine Learning in Accelerating the Discovery of Effective Natural Products

The rising worldwide challenge of viral hepatitis highlights the necessity for novel treatment approaches, as natural substances show encouraging prospects as antiviral agents. Improvements in analytical technologies and AI have revolutionized drug discovery, allowing for a more efficient identification of bioactive compounds from natural sources. Historically, compounds derived from plants have been crucial in addressing various diseases, serving as the foundation for numerous contemporary medications. Currently, the use of computational tools such as high-throughput screening, virtual modeling, molecular docking, and machine learning speeds up the discovery of potential lead compounds derived from natural products. More so, natural compounds such as hesperidin, quercetin, kaempferol, myricetin, and flavonoids have shown strong binding energies for hepatitis B surface antigen (HBsAg) [146]. AI-based methods, including molecular docking and quantitative structure–activity relationship (QSAR) models, enable scientists to examine extensive chemical databases, focusing on candidates that exhibit high effectiveness and low toxicity. This ability is especially important for HBV treatment, as recognizing effective, selective inhibitors is crucial for addressing drug resistance and enhancing therapeutic results [147]. 

AI and ML improve this process by tackling the issues of data complexity and the inconsistency of natural compounds [148,149]. By utilizing predictive modeling and deep learning techniques, researchers can enhance molecular profiling, minimizing false positives and boosting the accuracy of therapeutic candidates [150]. For instance, utilizing mass spectrometry (MS)-driven omics analyses alongside AI facilitates the comprehensive identification of functional molecules, offering an understanding of disease mechanisms and therapeutic targets [151]. These integrative approaches enhance the process from discovering natural compounds to preclinical evaluation, paving the way for the development of safer and more effective treatments for HBV. Additionally, this method corresponds with the wider global health aim of tackling infectious diseases by utilizing the chemical variety found in nature, bolstered by advanced computational and analytical techniques. The integration of conventional medicine, computational drug discovery, and AI signals a new phase in HBV treatment, encouraging innovation while maintaining the effectiveness and safety of upcoming therapies. 

## 10. Importance of Collaborative Research Between Computational Scientists, Pharmacologists, and Traditional Medicine Experts

Combining ethnopharmacology with bioinformatics offers a revolutionary method to enhance HBV treatment via joint research efforts. Traditional medicine provides a vast collection of natural substances with healing properties, but its clinical use necessitates a clear understanding of mechanisms and validation. Network pharmacology and computational methods, including molecular docking and cheminformatics, are crucial for elucidating the multitarget and multi-pathway interactions typical of traditional formulations. For example, methods such as system pharmacology and network analysis have clarified the mechanisms of action for intricate traditional formulas, uncovering potential drug candidates with multi-faceted efficacy. Joint initiatives among pharmacologists, computational scientists, and traditional medicine specialists can address deficiencies in data management, unify various databases, and enhance predictive models for compound–target relationships. This collaborative approach not only promotes the identification of new HBV treatments but also coincides with the increasing focus on personalized and precision medicines. 

Improvements in bioinformatics and AI-based approaches increase the capability of this partnership by boosting the effectiveness of natural compound screening and validation. Utilizing AI in network pharmacology enables thorough examination of intricate systems, such as discovering active compounds, charting target pathways, and accurately forecasting drug impacts [53,150]. Computational frameworks such as KNIME prediction methods, combined with in vitro and in vivo validation, facilitate an efficient pathway from discovery to therapeutic use [108]. For instance, functional components discovered via bioinformatics can be confirmed through experiments, as demonstrated in the clarification of antiviral actions within traditional medicinal formulations. Through promoting international cooperation, this unified research approach improves resource-sharing, reduces duplicative efforts, and speeds up the creation of affordable HBV treatments. Ultimately, these endeavors could transform the realm of antiviral drug development, providing accessible and efficient remedies for chronic hepatitis B while honoring the scientific and cultural value of traditional medicine. 

## 11. Potential Advancements for Predictive Modeling in HBV Therapy

The use of sophisticated in silico tools is transforming the field of HBV treatment research, facilitating the creation of predictive models that offer increased accuracy and dependability. Approaches like molecular docking, molecular dynamics simulations, and machine learning are now essential for discovering and characterizing active compounds for HBV treatment. For example, research on classical medicine mixtures such as Xiao-Chai-Hu-Tang (XCHT) employs these computational methods to discover bioactive substances like protoporphyrin and how they interact with targets like AKT1 and MAPK1 [17]. Utilizing these tools, researchers acquire knowledge about molecular interactions, evaluate draggability, and forecast safety profiles with impressive precision. Likewise, attempts aimed at the HBx viral protein using docking and simulation techniques have uncovered encouraging inhibitors like SC75741, demonstrating strong stability and interaction potential [152]. These developments emphasize the essential importance of in silico models in optimizing drug discovery workflows, lowering experimental expenses, and expediting therapeutic advancement. Further investigation reveals that in silico drug discovery methods offer an understanding of the mechanisms of action, reveal previously overlooked viral targets (including PreS1 Domain of HBsAg and cccDNA (Covalently Closed Circular DNA) Regulators), and facilitate the creation of specific inhibitors [6].

Additional advancements in predictive modeling, including the combination of QSAR and meta-based methods like Meta-iAVP, offer potential for enhanced HBV research [147,148]. QSAR models facilitate accurate mapping of molecular characteristics, which is evident in the discovery of flavonoids exhibiting anti-HBV activity via strong pharmacophore-driven screening and validation methods [149]. Conversely, machine learning-based meta-predictors such as Meta-iAVP improve the prediction of antiviral peptides by combining various feature sets with enhanced precision [147]. These tools not only broaden the range of possible therapeutic candidates but also guarantee the reproducibility and scalability of research conducted computationally. The future of HBV treatment research depends on the smooth combination of high-throughput computational models and experimental validation, creating a time of data-driven drug discovery with enhanced therapeutic effectiveness and safety profiles. 

Molecular docking techniques use algorithms like AutoDock and Glide to simulate interactions between ligands and target proteins, identifying optimal binding poses. Recent studies have successfully employed Auto Dock Vina and consensus docking to virtually screen a library of natural compounds against the hepatitis B virus (HBV) surface antigen, identifying compounds with significant binding affinities [153,154,155,156]. Free energy calculations using methods like Molecular Mechanics Poisson–Boltzmann Surface Area (MM-PBSA) and Molecular Mechanics Generalized Born Surface Area (MM-GBSA) estimate binding free energies by taking entropic and enthalpic contributions. These in silico methods typically yield values calculated in negative kilocalories per mole (kcal/mol), with more negative values indicating stronger binding energies. Binding free energy calculation is useful in demonstrating the efficacy of the in silico method for ligand-protein binding prediction [157]. Molecular docking’ predictions of binding affinities help prioritize which chemicals should be validated experimentally. Other studies have used in silico methods to identify natural compounds that exhibit antiviral properties against HBV, including curcumin interactions with HBV-polymerase via molecular docking and MD simulation. Molecular docking and MD simulation were carried out by applying AutoDock and GROMACS, respectively, to unveil curcumin’s effective binding at the active site of the protein (HBV-polymerase), displaying strong hydrophobic and hydrogen bonding. Curcumin’s inhibition of HBV-polymerase was further pharmacokinetically examined using the online SWISSADME, validating it as a potential lead compound [158,159]. More so, the antiviral potential of EGCG (a constituent of green tea that contains polyphenol) against HBV surface antigen (HBsAg) has been confirmed using in silico and in vitro methods, respectively. The application of Glide for molecular docking showed high binding energies of the EGCG–HBsAg complex, altering the surface antigen’s configuration and secretion. MD simulation was further employed to validate EGCG– HBsAg complex stability. Additional Pharmacokinetic validation showed advantageous absorption, distribution, metabolism, excretion, and toxicity properties, validating the usefulness of in silico methods in identifying and refining natural compounds for HBV therapy [158,159,160,161].

## 12. Overview of In Silico Successes and Failures in HBV Therapy and Clinical Translation of Computationally Repurposed Natural Compounds

Although several possible HBV inhibitors have been found using molecular docking and molecular dynamics (MD) simulations, only a few of them have advanced past in vitro validation. Their ineffective cellular absorption, limited solubility, and poor pharmacokinetic characteristics are the main obstacles to their clinical translation. These drawbacks highlight the necessity of additional optimization techniques to improve the bioavailability and therapeutic effectiveness of these natural compounds, including structural alterations, delivery methods based on nanocarriers, and prodrug formulations [162,163,164]. In preclinical and computational research, berberine, a naturally occurring alkaloid with significant antiviral qualities, has demonstrated encouraging outcomes for HBV treatment. Poor bioavailability, however, has impeded its clinical translation, requiring further approaches like delivery systems based on nanoparticles and structural alterations to maximize its therapeutic potential [165,166]. Similarly, resveratrol, which has been thoroughly investigated for its antiviral properties, has shown encouraging preclinical and in silico results but has not yet proven effective in HBV clinical studies. This is mainly because of its limited bioavailability and quick metabolism [166,167]. The evaluation of previous studies demonstrates the therapeutic significance of substances that have been studied for the treatment of HBV, including glycyrrhizin, silibinin, epigallocatechin gallate (EGCG), and silymarin [168,169,170,171,172]. Although silymarin and silibinin have shown antiviral effectiveness in HBV-infected individuals and have the potential to be used as adjuvant treatments, difficulties with dose optimization and bioavailability have limited their clinical use [173,174]. Another commonly used natural compound is glycyrrhizin. In traditional medicine, glycyrrhizin has shown clinical activity against HBV; nevertheless, to improve its bioavailability and therapeutic potential, additional formulation changes are required [175,176]. Although HBV drug repurposing has been successfully achieved through computational approaches, there is still considerable difficulty in turning these insights into clinical applications. 

## 13. Conclusions

The ongoing worldwide challenge of HBV, marked by considerable illness and death rates, highlights the pressing requirement for creative treatment approaches. Present antiviral treatments, such as nucleos(t)ide analogs and interferons, face challenges of drug resistance, insufficient viral suppression, and the failure to eliminate the covalently cccDNA reservoir. This has resulted in a fundamental change in drug discovery, where in silico techniques are becoming essential for the development and repurposing of therapeutic agents. Utilizing computational tools, researchers can speed up the discovery of new drug candidates, enhance their effectiveness, and lower the expenses and timelines linked to conventional drug development methods. In this situation, the incorporation of natural substances, with their diverse pharmacological characteristics, presents a hopeful path for tackling the challenges of HBV treatment. 

In silico methods like molecular docking, molecular dynamics simulations, and ADMET (absorption, distribution, metabolism, excretion, and toxicity) analysis have been crucial in clarifying the molecular mechanisms involved in HBV replication and pathogenesis. These computational methods have revealed important natural compounds, such as quercetin, epigallocatechin gallate, and curcumin, as possible multitarget inhibitors for HBV polymerase, cccDNA stability, and pathways for immune evasion. Additionally, sophisticated instruments such as network pharmacology and AI-based algorithms have improved our insight into the relationships between natural compounds and HBV targets, allowing for the forecasting of pharmacokinetic characteristics and toxicity profiles. These findings are crucial for the logical development of therapeutic agents that specifically target HBV pathways while also showing improved safety and effectiveness. 

The integration of computational techniques with conventional medicine and ethnopharmacological studies enhances the realm of HBV drug development. Natural remedies like Liujunzi and Xiaochaihu decoctions have shown promise in influencing HBV-related pathways, backed by molecular docking and dynamics simulations. These studies emphasize the collaboration between conventional knowledge and contemporary computational methods, establishing a basis for the creation of multitarget therapies. Additionally, the repurposing of current medications, directed by in silico approaches, provides an effective option for new drug development. By utilizing the recognized safety profiles and modes of action of existing compounds, researchers can hasten the progress of HBV treatments while reducing the risks linked to late-stage clinical failures. 

Despite these improvements, obstacles persist in fully harnessing the potential of in silico drug discovery and repurposing for HBV treatment. Concerns like data quality, model clarity, and converting computational forecasts into clinical effectiveness need to be tackled via thorough validation and cross-disciplinary cooperation. Nevertheless, combining computational chemical biology, bioinformatics, and AI with both traditional and modern pharmacological methods offers great potential. By adopting these approaches, the scientific community is more equipped to address the shortcomings of current treatments and lay the groundwork for safe, efficient, and scalable remedies for the HBV epidemic. 

However, acknowledging this present study’s limitations is important for future studies. Exclusively relying on an in silico approach is not adequate but requires in vitro and in vivo approaches to validate and confirm the experimental and biological mechanisms of new HBV drugs. More so, there are systematic flaws associated with in silico methods, such as approximating the molecular docking and molecular dynamics scores and affinity values. These limitations could be limited by integrating natural compounds and in silico, in vitro, and in vivo approaches in drug discovery in HBV therapeutics.

## Figures and Tables

**Table 1:** Comprehensive table summarizing drug repurposing and natural compounds in HBV therapy through in silico methods.

Drug/Compound Name and Structure	Original Indication/Compound Common Use	Target in HBV	Mechanism of Action	In silico Methods Used	Key Findings	Clinical/Preclinical Status	References
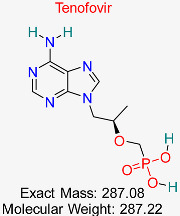	HIV	HBV Polymerase	Inhibits reverse transcription, reducing viral replication	Molecular docking, molecular dynamics simulation	High binding affinity with HBV polymerase; favorable ADMET profile	Approved for HBV treatment	[50,51]
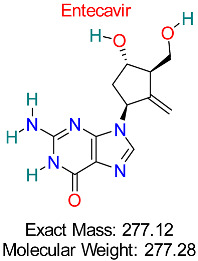	Herpes Simplex Virus	HBV Polymerase	Competitive inhibition of nucleotide incorporation during HBV replication	Molecular docking, ADMET analysis	Demonstrates strong selectivity and efficacy in HBV pathways	Approved for HBV treatment	[17,52]
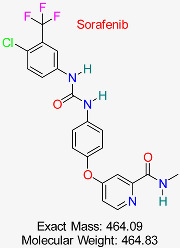	Hepatocellular Carcinoma	HBV cccDNA transcription	Inhibits tyrosine kinases, reducing cccDNA transcription and replication	Virtual screening, molecular docking	Identified potential off-target effects on HBV cccDNA pathways	Under investigation for HBV treatment	[53,54]
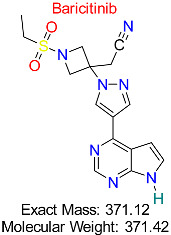	Rheumatoid Arthritis	HBV Immune Evasion Pathways	Janus kinase (JAK) inhibitor modulating immune responses to HBV infection	Molecular docking, molecular dynamics, AI modeling	High binding affinity to key immune evasion proteins; potential to enhance antiviral immunity	Preclinical stage	[55,56]
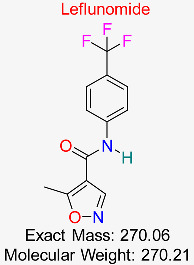	Rheumatoid Arthritis	HBV DNA Replication	Inhibits pyrimidine synthesis, disrupting HBV DNA synthesis	Docking, MD simulations, pharmacokinetics modeling	Favorable docking scores with HBV polymerase; potential to enhance existing HBV therapies	Preclinical stage	[57,58]
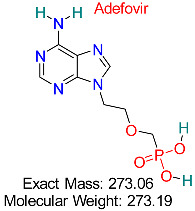	HIV	HBV Polymerase	Nucleotide analog that inhibits HBV polymerase activity	Structural modeling, molecular dynamics simulations	Effective against wild-type and resistant HBV strains	Approved for HBV treatment	[50,51]
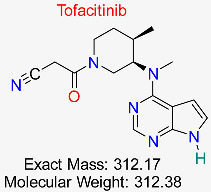	Rheumatoid Arthritis	HBV-Induced Inflammatory Pathways	Inhibits JAK-STAT pathway involved in HBV-induced liver inflammation	Molecular docking, network pharmacology	Strong inhibitory activity on inflammatory targets; favorable ADMET properties	Preclinical investigations	[57,59]
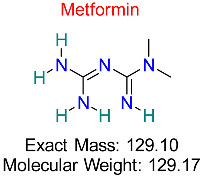	Type 2 Diabetes Mellitus	HBV cccDNA Regulation	Reduces cccDNA stability by modulating cellular energy metabolism	Molecular docking, ADMET analysis	Demonstrates potential to disrupt cccDNA stability in hepatocytes	Preclinical studies	[60,61]
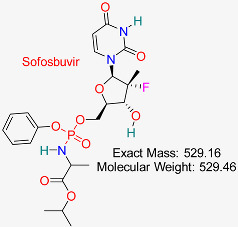	Hepatitis C Virus	HBV Polymerase	Inhibits HBV RNA-dependent RNA polymerase, disrupting replication	Molecular docking, dynamics, AI modeling	Effective binding to HBV polymerase; potential for combination therapies	Preclinical for HBV	[62,63]
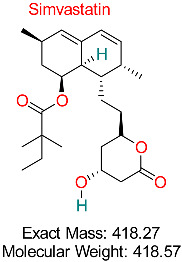	Hyperlipidemia	HBV Assembly	Reduces viral assembly and secretion by modulating cellular pathways	Docking, network pharmacology	Promising inhibition of HBV secretion pathways; synergistic effects with existing antivirals	Preclinical studies	[63,64]
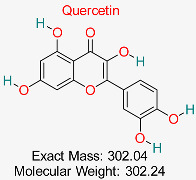	Natural flavonoid, possessing antioxidant, anti-inflammatory, antiviral, and anticancer effects, promoting cardiovascular wellness, neuroprotection, immune balance, and healing of wounds.	HBV cccDNA and Viral Polymerase	Inhibits key proteins involved in HBV replication and cccDNA maintenance	Molecular docking, dynamics simulations	Strong binding affinity to multiple HBV targets; multitarget action potential	Preclinical validation for HBV	[65,66]
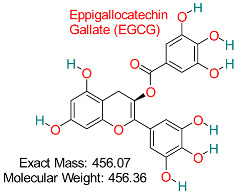	A potent catechin found in green tea, recognized for its antioxidant, anti-inflammatory, antiviral, and anticancer effects, aiding in heart health, brain performance, and immune system support.	HBV Viral Polymerase	Inhibits viral replication through polymerase targeting	Molecular docking, dynamics	High binding affinity to HBV polymerase with low toxicity	Preclinical studies	[67]
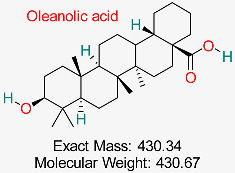	A natural triterpenoid exhibiting hepatoprotective, anti-inflammatory, antioxidant, and antiviral effects, commonly utilized for liver wellness and metabolic assistance.	HBV Assembly	Disrupts viral assembly and secretion pathways	Molecular docking, dynamics	Demonstrates effective HBV assembly inhibition; favorable pharmacokinetics	Preclinical investigations	[68]
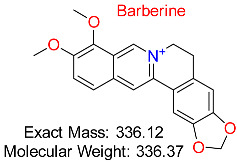	Natural alkaloid exhibiting antimicrobial, anti-inflammatory, antidiabetic, and cholesterol-reducing effects, frequently utilized to promote metabolic and cardiovascular well-being.	HBV DNA Replication	Interferes with viral replication by targeting HBV DNA synthesis	Virtual screening, molecular dynamics	Potent inhibition of HBV polymerase; synergistic potential with antivirals	Preclinical stage	[12]
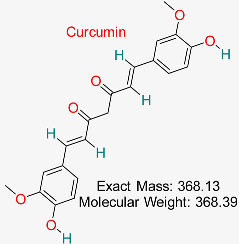	Bioactive substance present in turmeric possessing strong anti-inflammatory, antioxidant, and anticancer effects, commonly utilized for enhancing general well-being and controlling chronic illnesses.	HBV cccDNA Transcription	Modulates transcription factors involved in HBV cccDNA regulation	Network pharmacology, docking, dynamics	Identified as a multitarget inhibitor with low cytotoxicity	Preclinical studies	[67,69]
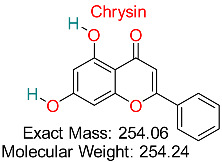	A natural flavonoid possessing antioxidant, anti-inflammatory, and anticancer characteristics, frequently studied for its possible benefits in treating anxiety, enhancing testosterone levels, and promoting cardiovascular wellness.	HBV Immune Modulation	Modulates immune pathways associated with HBV infection	Molecular docking, dynamics	Validated interactions with HBV immune evasion targets	Preclinical studies	[70,71]
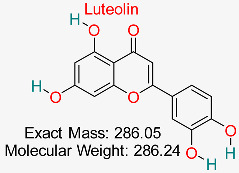	Flavonoid exhibiting strong antioxidant, anti-inflammatory, and anticancer effects, frequently researched for its involvement in neuroprotection, immune modulation, and lowering the risk of chronic diseases.	HBV Polymerase	Inhibits viral replication by targeting polymerase	Molecular docking, pharmacokinetics modeling	Demonstrates strong polymerase inhibition with favorable drug-likeness properties	Preclinical stage	[72]
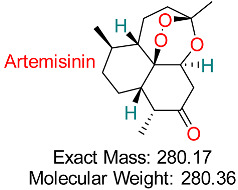	Derived from *Artemisia annua* (sweet wormwood), is widely known for its potent antimalarial properties and is also being explored for its anticancer, antiviral, and anti-inflammatory activities.	HBV Replication and Transcription	Targets viral replication pathways and transcription regulation	Docking, molecular dynamics	Promising multitarget activity against HBV; synergistic effects with other antivirals	Preclinical validation	[67]
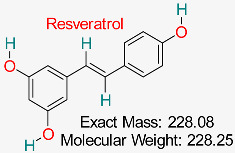	A natural substance present in grapes, berries, and peanuts; shows antioxidant, anti-inflammatory, cardioprotective, neuroprotective, and anticancer effects, with growing possibilities in antiviral treatment.	HBV cccDNA Stability	Reduces HBV cccDNA stability through modulation of host cellular factors	Molecular docking, dynamics, network pharmacology	High binding affinity to HBV cccDNA-related pathways; multitarget potential	Preclinical studies	[73,74]
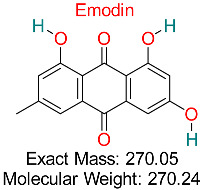	Naturally occurring anthraquinone compounds discovered in plants such as rhubarb and aloe vera exhibit anti-inflammatory, anticancer, antimicrobial, and antiviral effects, showing potential therapeutic uses for various illnesses, including HBV.	HBV Assembly and cccDNA Regulation	Inhibits HBV assembly and reduces cccDNA stability	Docking, ADMET analysis	Effective against multiple HBV targets; potential for combination therapies	Preclinical validation	[68]
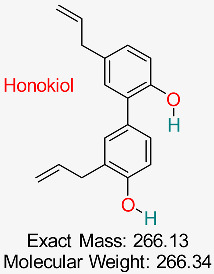	A biphenolic compound discovered in the bark and seeds of the Magnolia tree demonstrates multiple pharmacological effects, such as anti-inflammatory, antioxidant, anticancer, and antiviral characteristics, positioning it as a potential candidate for drug development, including treatment for HBV.	HBV Replication and Inflammation	Modulates inflammatory and viral replication pathways	Molecular docking, network pharmacology	Potential multitarget inhibitor; low toxicity	Preclinical studies	[67,75]
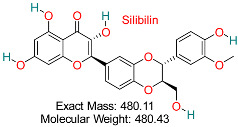	Flavonoids derived from milk thistle (*Silybum marianum*) have shown hepatoprotective, anti-inflammatory, antioxidant, and antiviral effects. It is recognized for its ability to block HBV replication and safeguard liver cells from harm, positioning it as a promising natural agent for HBV treatment.	HBV Replication and Oxidative Stress	Inhibits viral replication and modulates oxidative stress in liver cells	Docking, molecular dynamics, ADMET	Synergistic potential with standard antivirals	Preclinical validation	[68,72]
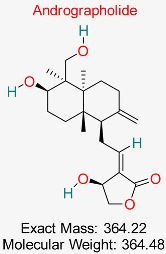	Diterpenoid lactone obtained from *Andrographis paniculata* is recognized for its anti-inflammatory, antioxidant, and antiviral capabilities. It has demonstrated promise in suppressing HBV replication, safeguarding liver cells, and regulating immune responses, positioning it as a potential option for therapeutic use in HBV therapy.	HBV Polymerase	Inhibits HBV polymerase activity	Molecular docking, dynamics	Potent HBV polymerase inhibition; favorable drug-likeness	Preclinical studies	[76,77]
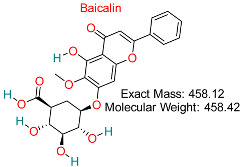	A flavonoid derived from *Scutellaria baicalensis* shows anti-inflammatory, antiviral, and liver-protective properties, possibly hindering HBV replication and liver injury.	HBV Replication	Disrupts viral replication pathways	Docking, dynamics, pharmacokinetics modeling	Promising activity against HBV replication	Preclinical investigations	[68,72]
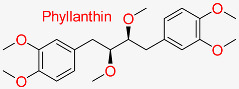	Lignan extracted from *Phyllanthus niruri* exhibits antiviral, hepatoprotective, and anti-inflammatory effects, especially against HBV, by suppressing viral replication and enhancing liver health.	HBV cccDNA Stability	Reduces stability of HBV cccDNA	Molecular docking, pharmacokinetics modeling	Effective multitarget activity against HBV pathways	Preclinical stage	[66]
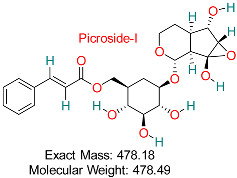	Key active ingredient from *Picrorhiza kurroa* shows hepatoprotective, anti-inflammatory, and antiviral effects, potentially blocking HBV replication and safeguarding liver cells from harm.	HBV DNA Replication	Disrupts HBV DNA replication pathways	Molecular docking, dynamics simulations	Favorable binding affinity to HBV DNA replication proteins	Preclinical studies	[78]
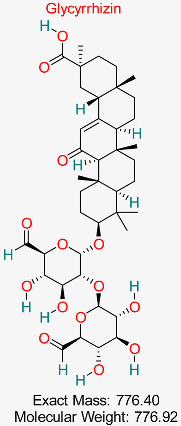	Extracted from *Glycyrrhiza glabra* (licorice root), it possesses strong antiviral, anti-inflammatory, and liver-protective effects, demonstrating promise in hindering HBV replication and enhancing liver function.	HBV Replication and Immune Modulation	Modulates immune response and inhibits viral replication	Molecular docking, network pharmacology	Identified as multitarget inhibitor with high safety profile	Preclinical validation	[67,68]
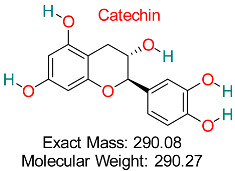	Flavonoids present in tea leaves exhibit antioxidant, anti-inflammatory, and antiviral effects, showing promise in blocking HBV replication and safeguarding liver cells from harm.	HBV cccDNA and Polymerase	Inhibits HBV replication and reduces cccDNA stability	Docking, dynamics simulations, ADMET analysis	Effective multitarget inhibitor against HBV pathways	Preclinical studies	[66,68,72]
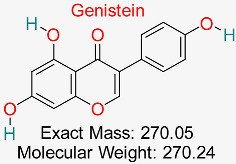	Isoflavonoid present in soybeans demonstrates antioxidant, anti-inflammatory, and antiviral abilities and has indicated promise in suppressing HBV replication and encouraging liver cell regeneration.	HBV Replication and Inflammatory Pathways	Modulates HBV replication and reduces inflammation	Docking, molecular dynamics	Strong binding to HBV replication proteins and inflammatory targets	Preclinical studies	[72]
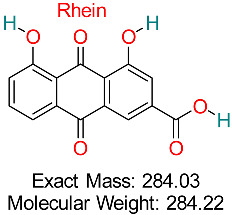	Anthraquinone derivatives discovered in a range of plants, such as *Rheum palmatum*, have shown anti-inflammatory, antioxidant, and antiviral effects and are being investigated for their ability to block HBV replication and alleviate liver damage.	HBV cccDNA Stability	Reduces HBV cccDNA by modulating cellular factors	Molecular docking, pharmacokinetics modeling	Promising multitarget potential for HBV therapy	Preclinical investigations	[79]
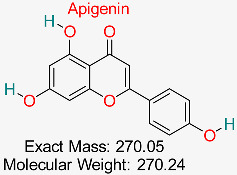	Flavonoids present in numerous plants like parsley, chamomile, and celery demonstrate antioxidant, anti-inflammatory, and antiviral effects, and research has examined their ability to hinder HBV replication and influence immune responses.	HBV Polymerase	Targets viral replication pathways	Molecular docking, network pharmacology	Demonstrates high binding affinity to HBV polymerase	Preclinical studies	[72,74]
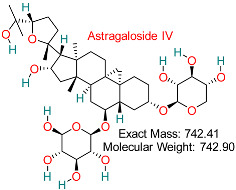	Saponin compounds obtained from *Astragalus membranaceus* have shown antioxidant, anti-inflammatory, and immune-enhancing effects and are being investigated for their ability to improve liver function and inhibit HBV replication.	HBV Immune Modulation	Modulates immune pathways related to HBV infection	Molecular docking, dynamics, pharmacokinetics modeling	Promising multitarget effects on immune evasion pathways	Preclinical studies	[80,81,82]
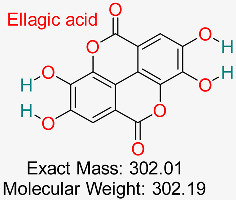	Polyphenolic compounds present in different fruits and vegetables have demonstrated potential as antiviral, anti-inflammatory, and antioxidant agents, and research is underway to assess their capacity to block HBV replication and safeguard liver cells from damage.	HBV Polymerase and cccDNA	Inhibits HBV replication and destabilizes cccDNA	Docking, dynamics, ADMET analysis	Effective multitarget inhibition of HBV pathways	Preclinical validation	[67,74]

## Data Availability

Not applicable.
